# Could ante-mortem computed tomography be useful in forensic pathology of traumatic intracranial haemorrhage?

**DOI:** 10.4102/ajlm.v10i1.1040

**Published:** 2021-07-29

**Authors:** Mmachuene I. Hlahla, Moshibudi J. Selatole

**Affiliations:** 1 Department of Forensic Pathology, Faculty of Health Sciences, University of Limpopo, Polokwane, South Africa

**Keywords:** forensic imaging, ante-mortem computed tomography, traumatic intracranial hemorrhage, forensic autopsy

## Abstract

**Background:**

Imaging techniques have proven valuable in forensic pathology practice, with computed tomography being preferred for forensic use. In the era of virtual autopsy and a low- to middle-income, resource-constrained country, a question arises as to whether ante-mortem computed tomography (ACT) could be cost-effective by reducing the number of invasive autopsies performed.

**Objective:**

The objective of this study was to assess the usefulness of ACT in forensic pathology by examining discrepancy rates between ACT scans and autopsy findings in cases of deceased individuals with traumatic intracranial haemorrhages and assess factors associated with discrepancies.

**Methods:**

Eighty-five cases of ACT and autopsy reports from 01 January 2014 to 31 December 2016 from the Polokwane Forensic Pathology Laboratory, South Africa, were analysed retrospectively. Using Cohen’s kappa statistics, measures of agreement and resultant discrepancy rates were determined. Also, the discrepancy patterns for each identified factor was also analysed.

**Results:**

The discrepancy rate between ACT and autopsy detection of haemorrhage was 24.71% while diagnostic categorisation of haemorrhage was 55.3%. Classification discrepancy was most observed in subarachnoid haemorrhages and least observed in extradural haemorrhages. A markedly reduced level of consciousness, hospital stay beyond two weeks and three or fewer years of doctors’ experience contributed to classification discrepancies.

**Conclusion:**

Ante-mortem computed tomography should be used only as an adjunct to autopsy findings. However, the low discrepancy rate seen for extradural haemorrhages implies that ACT may be useful in the forensic diagnosis of extradural haemorrhages.

## Introduction

Imaging techniques have in recent years been found to be greatly useful in forensic pathology.^1,2^ These modalities not only serve to augment but lower the invasive autopsies performed.^3^ The latter is convenient for various reasons, including religious and economic reasons. Computed tomography (CT) has been used as the preferred imaging modality in forensic imaging.^4^ Though in the era of virtual autopsies, the usefulness of ante-mortem computed tomography (ACT) compared to the inexpensive invasive autopsy in a middle-income country, such as South Africa, must be justified. Thus, it is necessary to determine whether ACT will be an augmentation or a cost-effective alternative to autopsy.

Head injuries are common, usually carry a high mortality rate, and are therefore important in forensic pathology practice. While post-mortem CT has been found useful in determining the cause of death in cases of traumatic intracranial haematomas,^2,5^ there is limited empirical evidence for the use of ACT scan findings to this effect.

Discrepancies between clinical or ante-mortem CT scan and autopsy findings exist and are common.^6^ According to Bruno et al.,^7^ radiologic interpretations cannot be programmed because interpretation is subject to a variety of factors such as doctors’ (radiologists’ and pathologists’) expertise and complex psychophysiological and cognitive level of the patient (e.g. level of consciousness). Thus, ACT interpretation errors are inevitable.

This study assessed the rate and pattern of discrepancies between ACT scan and conventional autopsy findings of intracranial haemorrhages in cases of traumatic head injury submitted for autopsy at a facility in a rural South Africa province.

## Methods

### Ethical considerations

Ethical clearance for the study was secured from Turfloop Research and Ethics Committee (TREC/268/2017: PG). Consent was not applicable as we were not working on human subjects but case reports, permission for which was obtained from the Limpopo Provincial Department of Health. The case files were coded with numbers and names of the deceased were not recorded.

### Study design

This quantitative descriptive study retrospectively analysed 85 cases of deceased individuals across three years from 01 January 2014 to 31 December 2016. Cases sustained head injuries, underwent ACT imaging and had no surgical intervention after being referred to an academic hospital. Deceased cases were subjected to autopsy procedure as per legal requirement at the attached forensic pathology facility in Limpopo, a rural province of South Africa.

### Data collection

Data were obtained from post-mortem reports from the forensic pathology laboratory while CT scan reports, clinical data and human resource records were obtained from the Pietersburg hospital records section and radiology department. Autopsy findings served as a reference point because studies have demonstrated that it remains superior to ACT scan for the detection of brain injuries.^1,8^ The majority (*n* = 80) of the cases had only one ACT scan done and the rest a second; only the first scan reports were therefore used in the study. The following intracranial haemorrhages were assessed from these reports: epidural, subdural and subarachnoid haemorrhages.

### Data analysis

Data were captured and analysed using Microsoft Excel (Microsoft Office Professional Plus 2013; Microsoft Corp., Redmond, Washington, United States) and International Business Machines Corporation Statistical Package for the Social Sciences version 23 (Armonk, New York, United States) running under Microsoft Windows (Microsoft Corp., Redmond, Washington, United States). Cross-tabulations were used to establish the percentage agreement between ACT scan and autopsy findings of extradural haemorrhage (EDH), subdural haemorrhage (SDH) and subarachnoid haemorrhage (SAH) single or combination occurrence. If there is agreement between the ACT scan and autopsy findings the individual case scored 1, but if there is a disagreement between the ACT scan and autopsy findings, the case scored 0. Levels of agreement and resultant discrepancy rates were determined using Cohen’s kappa statistics. The pattern of discrepancies for identified factors such as the level of consciousness by Glasgow Coma Scale, the length of hospital stay, the experience of the clinician (radiologist and pathologist) and the site of haemorrhage was also evaluated. Results were considered statistically significant when *p* was less than 0.05.

## Results

### Agreement in the detection and diagnosis of haemorrhages

In 75.29% (64/85) of cases, the ACT scan and autopsy agreed on the presence or absence of haemorrhage with a kappa coefficient of 0.3834 (Table 1). The remaining 24.71% represents the overall discrepancy rate between the ACT scan and autopsy detection of haemorrhage.

**TABLE 1:** Agreement between autopsy and ante-mortem computed tomography findings in the detection of intracranial haemorrhages, South Africa, 2014–2016.

Ante-mortem computed tomography findings		Autopsy findings		Kappa	Percentage agreement	*p*-value
		Present	Absent			
Overall (any haemorrhage)	Present	51	12	0.3834	75.29	< 0.001
	Absent	9	13			
**Types of haemorrhage**						
Extradural haemorrhage	Present	7	4	0.5823	90.59	< 0.001
	Absent	4	70			
Subdural haemorrhage	Present	26	7	0.4857	74.12	< 0.001
	Absent	15	37			
Subarachnoid haemorrhage	Present	31	19	0.3219	65.88	0.012
	Absent	10	25			

The highest agreement between the ACT scan and autopsy finding was recorded in the diagnosis of EDH (agreement 90.59%, kappa coefficient 0.5823 and discrepancy rate 9.41%) (Table 1). With regard to SDH, the agreement was 74.12% with a kappa coefficient of 0.4857 and a discrepancy rate of 25.88% (Table 1). The lowest agreement of 65.88% with a kappa coefficient of 0.3219 and the highest discrepancy rate of 34.12% was found for SAH (Table 1).

In 38 of the 85 cases (44.7%; 25 with and 13 without haemorrhage), both methods agreed in the diagnostic intracranial haemorrhage category (i.e. single, binary or ternary combinations of haemorrhages), denoting a diagnostic category discrepancy of 55.3% (Table 2 and Table 3). ACT agreed in 7 of the 11 cases categorised as singular SAH by autopsy (bolded) but reclassified three cases as having binary haemorrhage (SAH and SDH) and one case as absent (no haemorrhage). For 25 cases classified as ‘Absent’ (having no haemorrhage) by autopsy, ACT agreed in 13 (bolded) cases but categorised the remaining 12 as having different haemorrhages.

**TABLE 2:** Summary of diagnostic category for intracranial haemorrhage, South Africa, 2014–2016.

Radiology findings	Autopsy findings	
	Haemorrhage diagnosis	No haemorrhage diagnosis
Agree	25	13
Disagree	35	12

**TABLE 3:** Summary of misclassification of detection category agreement and discrepancies observed for haemorrhages, South Africa, 2014–2016.

Radiology finding	Autopsy findings								Total
	EDH	EDH/SAH	EDH/SDH	EDH/SDH/SAH	SAH	SDH	SDH/SAH	Absent	
EDH	**2**	-	-	-	-	-	-	-	2
EDH/SAH	1	**1**	-	-	-	-	-	2	4
EDH/SDH	1	-	-	1	-	-	1	-	3
EDH/SDH/SAH	-	-	-	**1**	-	-	1	-	2
SAH	-	2	-	-	**7**	3	5	7	24
SDH	-	-	-	-	-	**3**	3	2	8
SDH/SAH	-	-	1	-	3	4	**11**	1	20
Absent	1	-	-	-	1	3	4	**13**	22
**Total**	**5**	**3**	**1**	**2**	**11**	**13**	**25**	**25**	**85**

Note: Bold data – Agreement in diagnostic category for intracranial haemorrhage (singly or combinations); Non-bold data – Discrepancies in diagnostic category for intracranial haemorrhage (singly or combinations).

EDH, extradural haemorrhage; SDH, subdural haemorrhage; SAH, subarachnoid haemorrhage.

### Pattern of discrepancies with regard to identified factors

**FIGURE 1:**
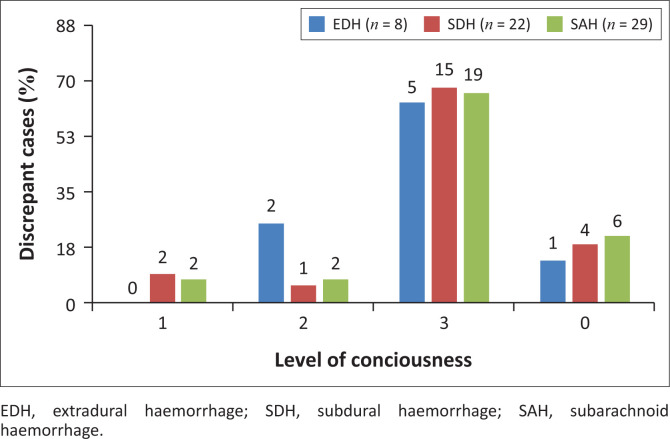
Discrepancies and level of consciousness, South Africa, 2014–2016. 1 = mild (GCS 15–13), 2 = moderate (GCS 12–9), 3 = severe (GCS ≤ 8), 0 = unspecified.

The analysis revealed that most discrepant intracranial haemorrhage diagnoses (5 out of 8 cases for EDH; 15 out of 22 cases for SDH; 19 out of 29 cases for SAH) were seen in patients with markedly low levels of consciousness, denoting severe traumatic head injury (Figure 1).

**FIGURE 2:**
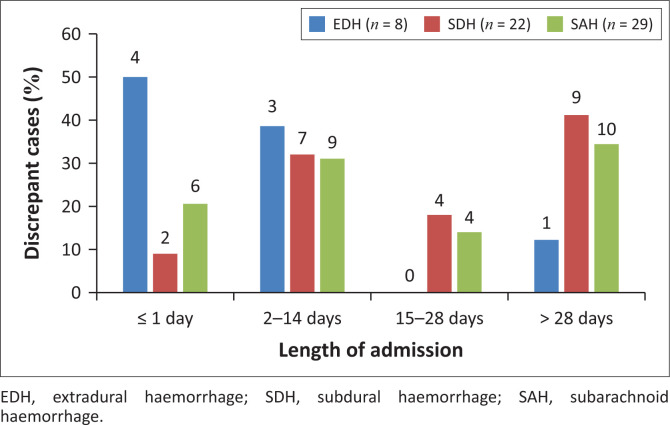
Discrepancies and length of admission, South Africa, 2014–2016.

Half (4/8) of the discrepant cases for EDH were found to have been admitted on average for a day or less (Figure 2). On the other hand, most discrepancies for SAH were seen in cases that were admitted for two weeks or less (9/29) and four weeks or more (10/29) on average, while for SDH the majority (9/22) stayed in the hospital for more than a month.

**FIGURE 3:**
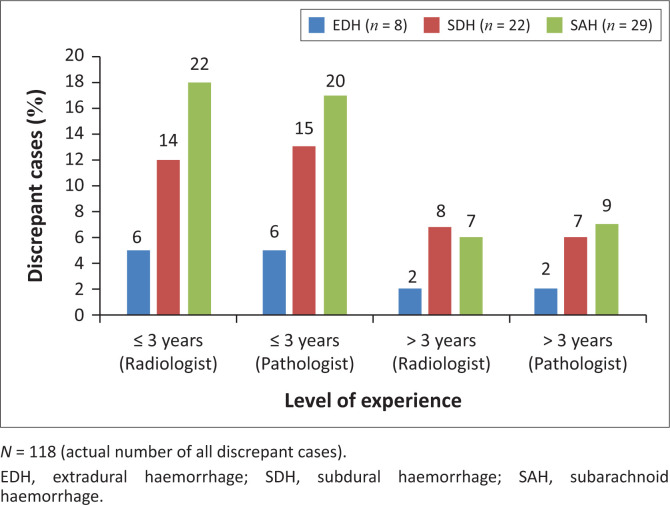
Discrepancies and level of experience (in years), South Africa, 2014–2016.

In addition, more discrepant case diagnoses were seen with a radiologist or pathologist who had less than three years of working experience (Figure 3).

## Discussion

The current study found a significant difference in haemorrhage detection between ACT and autopsy. Individual intracranial haemorrhage detection discrepancies ranged from 9.41% to 34.12%, with SAH carrying the highest rate. This is consistent with what has been reported by a number of previous studies that collectively reveal discrepancy rates ranging from 9% to 39%.^5,9^ There are varying findings concerning discrepancy rates for SAH with the majority of previous studies showing high discrepancy^10,11,12^ in keeping with the current study.

The study also found that in general ACT had a diagnostic accuracy of 44.7%. As such, the high level of discrepancy for SAH was attributable to misclassification, which may mean misdiagnosis, and to a combination of haemorrhages that could have masked the SAH. Also, a high SAH discrepancy rate (79% of SAH cases) was noted with prolonged length of hospital stay, probably due to haemorrhage resorption with time. A progressive clearance of red blood cells in the cerebrospinal fluid results in approximately 50% of SAHs not being visualised after one week of occurrence,^13,14^ and this process can be shorter or longer.^15^ This implies that the majority of the SAH previously diagnosed under an ACT scan may after approximately three weeks not be seen in an autopsy if the patient demises. This is dependent on the amount of SAH in the leptomeninges. This was evident from the current study where the ACT scan superseded autopsy in the diagnosis of SAH, which was consistent with previous studies.^11,14^

The high level of agreement of agreement (90.59%) observed for EDH diagnosis (with high true negative rate) was corroborated in a similar study comparing ACT and autopsy findings^11^ and when comparing post-mortem CT diagnoses to autopsy results.^9^ We opine that the high level of discrepancy in cases admitted for a day or less may be because it takes more than a day for the typical shape of the haemorrhage to appear.

More discrepancies were noted in cases with markedly low levels of consciousness, denoting severe injuries. This may be because critically ill patients may not assume specific positions required to visualise the haemorrhage, particularly when the haemorrhage is small in size, a notion also suggested by Liisanantti and Ala-Kokko.^5^

Practitioners (radiologists and pathologists) with a low level of experience made more discrepant diagnoses. Although most studies support that experience appears to decrease discrepancies,^16,17^ they also agree that there are multifactorial and complex factors that can result in those discrepancies. Some stipulate that the main reason for the discrepancies in lower postgraduate trainees has been identified as a lack of knowledge.^18,19^ Doctors with low levels of experience are typically registrars and junior medical officers. Lee et al.^20^ report that discrepancies tend to be higher among registrars, owing to ‘physical discomfort, eye strain and lack of motivation’, which intensify by the end of the workday. Moreover, registrars and medical officers work overtime, during which they have to work overnight. This may result in focusing difficulty and hence reduced diagnostic or detection accuracy.^21,22^

Length of admission has also been shown to affect agreement for SDH. The study showed that the greater part of the discrepant SDH cases were seen in patients who were admitted for more than a month on average. A possible reason could be the effects of the healing processes, which can happen in thinner blood collection cases such that after a month the haemorrhage may be enclosed or completely absorbed.^23,24^

Brain slicing intervals during autopsies are usually not the same as the intervals used during ACT imaging and this may also account for the general discrepancy rate. Delayed intracranial haemorrhage could occur after the ACT scan is obtained and could also account for the discrepancies as most of the cases did not undergo a subsequent ACT scan. A useful scenario to study this would have been to have the ACT and autopsy on the same day.

### Limitations

The major limitation of this study is the small sample size. Moreover, the study addressed only three types of brain haemorrhages (EDH, SDH and SAH); therefore, the findings cannot be generalised to forensic pathology practice, or any other type of intracranial haemorrhage. Further research with a larger sample size and broader scope is recommended.

### Conclusion

The overall detection discrepancy rate of 24.74% and CT diagnostic accuracy of 44.7% implies that ACT scans may not be used as an altervative to reduce the number of autopsies performed at the mentioned facility but can only be used as an adjunct to autopsy. However, the low discrepancy rate in EDH, especially after a day of admission, implies that ACT may be useful for the diagnosis of this haemorrhage in the forensic setting. A markedly reduced level of consciousness, length of hospital stay depending on the type of haemorrhage and three or fewer years of doctors’ experience all contributed to discrepancies observed between ACT and autopsy findings. The study employed a limited sample and thus calls for more similar studies in both high and low- and middle-income countries.
